# Enhanced Bioactive Coffee Cherry: Infusion of Submerged-Fermented Green Coffee Beans via Vacuum Impregnation

**DOI:** 10.3390/foods14071165

**Published:** 2025-03-27

**Authors:** Pipat Tangjaidee, Sukan Braspaiboon, Naphatsawan Singhadechachai, Suphat Phongthai, Phatthanaphong Therdtatha, Pornchai Rachtanapun, Sarana Rose Sommano, Phisit Seesuriyachan

**Affiliations:** 1 Faculty of Agro-Industry, Chiang Mai University, Chiang Mai 50100, Thailand; pipat.t@cmu.ac.th (P.T.); sukan.bras@cmu.ac.th (S.B.); naphatsawan_sing@cmu.ac.th (N.S.); suphat.phongthai@cmu.ac.th (S.P.); phatthanaphong.th@cmu.ac.th (P.T.); pornchai.r@cmu.ac.th (P.R.); 2 Center of Excellence in Agro Bio-Circular-Green Industry (Agro BCG), Chiang Mai University, Chiang Mai 50100, Thailand; sarana.s@cmu.ac.th; 3 Department of Plant and Soil Science, Chiang Mai University, Chiang Mai 50200, Thailand; 4 Advanced Technology and Innovation Management for Creative Economy Research Group (AIMCE), Department of Industrial Engineering, Faculty of Engineering, Chiang Mai University, Chiang Mai 50200, Thailand

**Keywords:** submerged fermentation, ultrasound treatment, fermented green coffee bean, coffee cherry utilization, bioactive compound infusion

## Abstract

Submerged fermentation offers a controlled environment for coffee processing, ensuring a consistent temperature and aerobic–anaerobic conditions, making it a superior alternative to solid-state fermentation. This study aimed to optimize submerged fermentation conditions for green coffee beans to maximize total phenolic content (TPC) and antioxidant activity, such as ABTS (2,2′-azino-bis(3-ethylbenzothiazoline-6-sulfonic acid), DPPH (2,2-Diphenyl-1-picrylhydrazyl), and FRAP (the ferric reducing antioxidant power). Additionally, pH, yeast, and lactic acid bacteria counts were monitored. Fermentation was conducted with selective microbial starters, a varying temperature (25–35 °C), incubation time (3–9 days), and coffee weight (5–10 g) using a Box–Behnken design. To enhance bioactive compound infusion, fresh coffee cherries underwent ultrasonic treatment, increasing their porosity and water-holding capacity. Vacuum impregnation was then used to infuse fermented green coffee bean extract into the cherries. The lowest pH coincided with peak yeast growth, while the coffee weight significantly influenced all responses. The incubation time affected most parameters except DPPH activity, and the temperature impacted only ABTS and DPPH activities. Optimal conditions (35 °C; 7.21 days; 10 g) yielded a TPC of 480.25 µmol GAE/100 g with ABTS, DPPH, and FRAP activities of 725.71, 164.15 and 443.60 µmol TE/g, respectively. Ultrasound-treated coffee cherries exhibited increased porosity and absorption capacity, facilitating enhanced bioactive compound infusion during 3 h of vacuum impregnation. In conclusion, submerged fermentation effectively improves bioactive compound production, while ultrasound treatment and vacuum impregnation present promising methods for developing high-value dehydrated coffee cherry products.

## 1. Introduction

Today, the requirement for functional foods and beverages has increased because people are more aware of health issues and need foods that provide health advantages beyond basic nutrition. One of the world’s most demanded drinks is coffee, containing impressive contents of antioxidants and phenolic compounds [1]. Furthermore, the consumption of caffeinated coffee is related to diminishing the risk of heart failure from type 2 diabetes [2,3]. In addition to its health benefits, coffee is also consumed for people to relax and enjoy its taste. Accordingly, the process of coffee production must be meticulous to meet the drinker’s expectations. 

Fermentation is a significant process to modify phenolic compounds and other metabolites, which in turn impacts the unique aroma and flavor of specialty coffees [4]. A fermentation process was initiated by removing mucilage layers of coffee cherries ahead of a drying step to acquire green coffee beans [5]. Afterward, green coffee beans were fermented to elevate levels of antioxidant activities and phenolic compounds [6]. Green coffee beans are sources rich in antioxidant content and phenolic compounds. Their major phenolic compounds are chlorogenic acids and caffeic acids, potent for a range of health benefits, such as carcinogenesis, hepatocarcinoma, and mutagenesis [7,8,9]. Moreover, green coffee beans contain numerous volatile and semi-volatile chemicals, such as alcohols, acids, esters, aldehydes, ketones, and furans. These molecules are crucial for forming fragrance and flavor during a roasting process [10].

Submerged fermentation is the condition in which water is added to the fermentation process, influencing microbial growth. Water-adding conditions can be categorized into two categories: solid-state and submerged fermentation. Microorganisms can grow depending on moisture from whole coffee cherries during solid-state fermentation, while they can grow in the liquid state of submerged fermentation. Submerged fermentation provides water activity and decreases oxygen availability. Therefore, this method promotes the growth of bacteria superior to fungi, including the speed of mucilage removal. Moreover, this technique can control the species diversity and growth rate of microorganisms, aerobic or anaerobic conditions, and the consistency of heat transfers [4]. Although submerged fermentation is obsolete, it is also beneficial for coffee commercialization. This method can control the fermentation parameters, influencing specialty coffee regarding metabolic and bioactive compounds and customer acceptance due to different sensory profiles derived from this technique [4]. Apart from awakening, antioxidant capacity is one of the most bioactive activities consumers require from drinking coffee. The methods of antioxidant quantification have been numerous because a single method cannot determine all antioxidants. The general methods for antioxidant analysis are total phenolic content (TPC) and antioxidant activity, such as ABTS (2,2′-azino-bis (3-ethylbenzothiazoline-6-sulfonic acid), DPPH (2,2-Diphenyl-1-picrylhydrazyl), and FRAP (the ferric-reducing antioxidant power) [4].

Microbial species are another crucial element resulting in different specialty coffees. Fermentation, depending on the sole epiphytic microbiota present in coffee beans, cannot control quality consistency and favorable chemical and biological characteristics. Epiphytic microbiota species—natural microorganisms found in green coffee beans—are lactic acid bacteria (LAB), acetic acid bacteria, enterobacteria, and yeasts [11,12,13]. Thus, they were produced as a single starter to investigate the developed profiles of flavors and metabolites of Arabica coffee fermentation from individual species [14,15]. Using a starter with yeast was indicated as having the potential to improve coffee quality at lower altitudes of solution. In addition, yeasts also generate a velvet-like body perception and caramel-like coffee taste. Meanwhile, lactic acid bacteria are another significant species for submerged fermentation. They increase volatile aroma compounds, leading to consumer satisfaction [4]. Recently, the combination of microbial cocktail starters has been applied to coffee fermentation [16] because a single-species starter might not satisfy consumers, who prefer a complexity of flavor and taste. However, the inoculation of microbial combinations is still rare for coffee fermentation, especially submerged fermentation.

Ultrasound, a non-thermal food processing technology, operates with sound waves at 20–100 kHz and generates effects such as cavitation, fluid mixing, and shear forces. It offers benefits in food preservation, mass transfer, texture alteration, and food analysis while assisting thermal treatments [17]. In a previous study, ultrasound pretreatment of peach slices degraded the cell wall, increased the elastic modulus, and enhanced the pore structure and porosity, leading to a crisper texture. The presence of microchannels reduced the solids content, resulting in softer peach samples with lower hardness values [18].

Based on this information, this research aims to optimize the submerged fermentation conditions of temperature, incubation time, and coffee weight of green coffee beans to receive the maximum TPC content and antioxidant activities. In addition, the growth of yeasts and LAB, including pH in each fermentation condition, was also investigated.

## 2. Materials and Methods

### 2.1. Green Coffee Beans and Chemicals

Green Robusta coffee beans and cherry were derived from the Wa Wi sub-district, Mae Suai district, Chiang Rai, Thailand, located at 1.2 km above sea level (location: 19.842581704907417, 99.4981672927493). After harvesting, the green coffee beans were produced from fresh coffee cherry using a dry process by sun heat until their moisture contents were about 7–12% dry basis. Afterward, the dried coffee beans were dehulled in order to get rid of the pulp. Then, the green coffee beans were kept and transported in the dark at 30 °C for approximately 3 h and subsequently stored at −20 °C to maintain quality.

The coffee beans were prepared by defrosting at room temperature, followed by air drying at 45 °C for 24 h. The dried coffee beans were cooled at room temperature in a desiccator. The green coffee beans were finely ground and separated by a 100-mesh sieve (100–200 µm) prior to the fermentation process.

Chemical reagents comprising gallic acid, 2,2′-azino-bis (3-ethylbenzthiazoline-6-sulphonic acid) (ABTS), 2,2-Diphenyl-1-picrylhydrazyl (DPPH), and 6-hydroxy-2,5,7,8-tetramethylchroman-2-carboxylic acid (Trolox) were derived from Sigma Aldrich (St. Louis, MO, USA).

### 2.2. Collection of Microbial Culture

Four yeast strains consisting of *Hanseniaspora osmophila* TISTR 6031, *Hanseniaspora vineae* TISTR 6032, *Schizosaccharomyces osmophilus* TISTR 6033, and *Saccharomyces cerevisiae* TISTR 6034 were cultured in a yeast mold (YM) broth containing 5 g/L animal-tissue peptic digest, 3 g/L yeast extract, 3 g/L malt extract, and 10 g/L dextrose (HiMedia, Maharashtra, India), and the pH was approximately 6.2. The YM broth was incubated at 150 rpm of shaking speed at 30 ◦C for 36–48 h. In contrast, *Lactobacillus plantarum* TISTR 2265 was cultured in de Man, Rogosa, and a Sharpe (MRS) broth containing 10 g/L proteose peptone, 10 g/L beef extract, 5 g/L yeast extract, 20 g/L dextrose, 1 g/L polysorbate, 2 g/L C_6_H_14_N_2_O_7_, 5 g/L C_2_H_3_NaO_2_, 0.1 g/L MgSO_4_, 0.05 g/L MnSO_4_, 2 g/L K_2_HPO_4_ (Difco, Bordeaux, France). The MRS broth was adjusted to a pH of 6.2 and incubated at 37 °C for 24 h. The microbial culture was stocked with 20% (*v*/*v*) glycerol at −20 °C until use.

### 2.3. Preparation of Starters

Yeast cultured in yeast malt dextrose (YMD) broth was inoculated at 10% (*v*/*v*) of microbial culture into 100 mL of YMD. The yeast inoculum was incubated at 30 °C with shaking at 150 rpm for 24 h or until its optical density (OD) was one. Meanwhile, the bacterium cultured in the MRS broth was inoculated at 10% (*v*/*v*) of microbial culture into 100 mL of MRS broth. The bacterial inoculum was incubated at 37 °C for 24 h or until its OD was one before being used in the fermentation process.

### 2.4. Submerged Fermentation

The coffee powder was weighed according to Table S1 into 90 mL of deionized water, while a sterilized sucrose solution was supplemented with 14.25% *v*/*v*. Microbial starters derived from 2% of each microbial strain were inoculated into the slurry by 10% of the total volume. Submerged fermentation was shaken at 50 rpm for the first 24 h and 100 rpm until the due time. The temperature and time of the submerged fermentation were according to Table S1. After fermentation, the fermented green coffee beans (FGCBs) were dried at 45 °C for 24 h before the FGCBs were extracted by a maceration technique with 10 folds of 70% ethanol at 30 °C for 24 h. Then, the FGCBs were evaporated at 30 °C until the volume was reduced by 10fold to a concentrate. Finally, the FGCB extracts were further analyzed for antioxidant activities and impregnated into the coffee cherries.

### 2.5. Optimization of Submerged Fermentation

Three variables, the temperature (X_1_), incubation time (X_2_), and the weight of coffee powder (X_3_), were analyzed for individual influence and their interactive effects. The 33 experimental conditions of submerged fermentation were generated according to a Box–Behnken design (Table S1). Responses were measured in triplicate of TPC contents and antioxidant activities, consisting of ABTS activity, DPPH radical scavenging activity, and FRAP. In addition, the pH value and microbial count of yeasts and LAB were also measured. The response surface methodology was applied to fit a polynomial equation to the experimental result.

A quadratic polynomial model was fitted to the experimental data, obtaining a regression coefficient. The generalized quadratic model employed in the response surface analysis is the following Equation (1).

(1) $$Y=\beta_{0}+\sum_{i=1}^{3}\beta iXi+\sum_{i=1}^{3}{\beta iiXi}^{2}+\sum_{i=1}^{2}\sum_{i=2}^{3}\beta ijXiXj$$

where *β_0_* is the constant, *βi* is the linear coefficient, *βii* is the quadratic coefficient, and *βij* is the interaction coefficient. *Xi* and *Xj* are the levels of the independent variables.

The analysis of variance (ANOVA) table contained the effect and regression coefficients for each individual linear, quadratic, and interaction term. The F-value was assessed at a 0.05 probability to statistically assess the significance of each term in the polynomial. Statistical computation based on the regression coefficient was used to create three-dimensional contour plots from the regression models.

### 2.6. Ultrasonic-Assisted Treatment of Coffee Cherry

The coffee cherry was submerged in distilled water at a ratio of 1/5 (*w*/*v*) and treated with ultrasonic waves at 350 W and 550W for 10 to 30 min. The physical properties of the treated coffee cherry, including water-holding capacity, hardness, and the morphology of the structure observed by scanning electron microscopy (SEM), were detected.

### 2.7. Vacuum Impregnation

Ultrasonic-treated coffee cherries were immersed in a 50% (*w*/*v*) sugar solution containing the FGCB extract at a concentration of 5 mg/mL. Vacuum impregnation was performed using a vacuum oven (Binder 9030-0031, Cole-Parmer, Germany) with alternating vacuum–pulse and release cycles every 30 min for a total duration of 1 to 5 h. The efficiency of vacuum impregnation in infusing the FGCB extract into the coffee cherries was assessed by analyzing the total phenolic content, antioxidant activity, and chlorogenic acid concentration.

### 2.8. The Measurements of Microbial Growth and pH

The green coffee beans, fermented by the starter according to Table S1, were diluted with phosphate buffer saline at a 10-fold dilution until they reached a 10^−6^ dilution. The 10^−1^ to 10^−6^ dilutions of LAB and yeasts were quantified. Yeasts were quantified on yeast malt dextrose agar. Meanwhile, LAB was determined by drop plate technique on MRS media fortified by calcium carbonate and 1% agar. The microbial count was expressed in terms of a log colony-forming unit (cfu)/g sample.

### 2.9. TPC Determination

The TPC content was determined according to a Folin–Ciocalteu assay [19]. An aliquot (0.5 mL) of the FGCB extract was mixed with 0.5 mL of Folin–Ciocalteu reagent. Afterward, 2 mL of 10% (*w*/*v*) Na_2_CO_3_ and 9.5 mL of distilled water were mixed together into the previous mixture and incubated in the dark for 30 min (ambient temperature). The absorbance of the solution was measured at 765 nm using a spectrophotometer (BioMate 3, Thermo Scientific, Waltham, MA, USA). The TPC content was reported as a gallic acid equivalent (mg GAE/100 g extract, dry weight) and computed by a calibration curve of gallic acids, ranging from 0 to 100 μg/mL (R^2^ = 0.998).

### 2.10. Determination of Antioxidant Activities

#### 2.10.1. ABTS Assay

The ABTS assay was conducted according to the method of Marc et al. [20] with some modifications. The FGCB extract (0.1 mL) was mixed with 10 mL of ABTS working solution and incubated in the dark for 6–7 min (ambient temperature). Afterward, the absorbance of the solution was measured at 734 nm using a spectrophotometer. The ABTS activity was expressed in Trolox equivalents (μmol TE/g extract, dry weight), determined using a Trolox calibration curve ranging from 0 to 0.6 μmol/mL (R^2^ = 0.993).

#### 2.10.2. DPPH Radical Scavenging Assay

The DPPH assay was conducted following the procedure of Arulpriya et al. [21] with slight modifications. A 0.5 mL of the FGCB extract was mixed with 0.1 mM DPPH solution (1 mL) and incubated in the dark for 30 min at an ambient temperature. The absorbance was measured at 517 nm using a spectrophotometer. The antioxidant activity was expressed as μmol TE/g extract, dry weight, estimated by 0–0.25 μmol/mL of Trolox calibration (R^2^ = 0.994). The percentage of DPPH inhibition was calculated using Formula (2).

DPPH inhibition (%) = (A_control_ − A_sample_)/A_control_ × 100 (2)

where A_control_ presents the absorbance of the control reaction (DPPH solution), and A_sample_ is the absorbance of the submerged–fermentation solution.

#### 2.10.3. FRAP Assay

The FRAP assay was performed according to the procedure explained by Li et al. [22] with a few modifications. After submerged fermentation, 0.1 mL of the FGCB extract was blended with the FRAP working solution (3 mL) and darkly incubated at 37 °C. Afterward, the absorbance was measured at 593 nm using a spectrophotometer. The FRAP activity was reported as μmol TE/g extract, dry weight, computed based on a 0–0.6 μmol/mL of the Trolox calibration curve (R^2^ = 0.9949).

### 2.11. Water-Holding Capacity (WHC) of Coffee Cherry

The WHC of ultrasonic-treated coffee cherry was determined according to [23]. Briefly, the sample (1 g) was mixed with 10 mL of distilled water, then vortexed for 1 min, centrifuged at 3000× *g* for 30 min, and the volume of the supernatant was determined. The WHC was expressed as grams of water held per gram of sample.

### 2.12. Hardness Texture of Coffee Cherry

The hardness of ultrasonic-treated coffee cherry was measured. The compression plate was assessed by using a texture analyzer (TA-XT Plus, Stable Micro Systems, London, UK) equipped with a 5 kg load cell and compression probe (T-Cy 36) with a speed of 0.5 mm/s and a distance prolongation of 20 mm. The force at the first major drop in the force–deformation curve (Fmax) and deformation at maximum force were obtained in duplicate for two samples of each bread. The results of hardness are expressed as force (N). 

### 2.13. Scanning Electron Microscopy (SEM) of Coffee Cherry

A scanning electron microscope (SEM) (FE-SEM, Tescan Clara, Brno, Czech Republic) was used to observe the microstructures of coffee cherries after ultrasonic treatment. Before SEM analysis, samples were lyophilized and mounted on specimen stubs. The mounted coffee cherry samples were coated with platinum-palladium (Pt-Pd), and a cross-section of the noodles was observed with the SEM at 300× magnification. 

### 2.14. Chlorogenic Acid Determination

Chlorogenic acid analysis was performed in the LC-MS (Agilent Inc., Santa Clara, CA, USA). The operation of liquid chromatography was conducted using RP column (Poreshell 120 EC-C18 column (4 µm, 4.6 × 150 mm; Agilent, St. Louis, MO, USA). The gradient elution was performed using a solvent system. Solvent system A was water containing 0.1% formic acid, while solvent system B was acetonitrile containing 0.1% formic acid. A 0.8 mL/min flow rate was applied for the gradient chromatographic separation at 30 °C. The gradient condition followed the following sequence: 95% (A):5% (B), 2 min; 70% (A):30% (B), 15 min; 30% (A):70% (B), 5 min; 95% (A):5% (B), 2 min. The FGCB extract was dissolved in a mixture of acetonitrile and water (1:1 *v*/*v*), passed through a 0.22 µm filter, and injected at a volume of 10 μL. The signals of the separated compounds were recorded at 254 nm and using the accurate masses at 353 *m*/*z* in a negative charge ion.

### 2.15. Statistical Analysis

All experiments were expressed by mean ± standard deviation (SD) in triplicate. One-way ANOVA was employed to analyze the significance of the regression model. Significant differences between the means were measured using a Duncan’s multiple range test (*p* ≤ 0.05). The statistical analysis, including data analysis, experimental design, model generation, and response surface plots, was performed using Design Expert 6.0.10 software (Stat-Ease, Minneapolis, MN, USA).

## 3. Results and Discussion

### 3.1. Microbial Count and pH

The microbial count and pH of the submerged fermentation of green coffee beans is presented in Table S1. The lowest count of total yeast was 3.42 log cfu/g, observed at a temperature of 25 °C, an incubation time of 6 days, and 5 g of coffee weight. In contrast, the maximum count of total yeast was 5.84 log cfu/g, during which incubation time and coffee weight were increased to 9 days and 7.5 g, respectively. Meanwhile, the lowest and highest LAB counts were 3.39 log cfu/g (35 °C, 9 days of incubation, 5 g coffee) and 5.79 log cfu/g (30 °C, 3 days of incubation, 10 g coffee), respectively. The pH value was minimal at 3.3, the same condition as the maximum total yeast count. On the other hand, the highest pH value was 4.94 at a temperature of 25 °C, an incubation time of 3 days, and 7.5 g of coffee weight. The ANOVA analysis reported that the incubation time significantly affects the LAB count and pH (*p* < 0.05), while the temperature and coffee weight impact only the LAB and yeast counts, respectively.

The lowest pH value corresponded to the maximum yeast count. This result was similar to the pH of coffee cherries fermented by native yeasts, such as *Wickerhamomyces anomalus*, *S. fibuligera*, and *S. cerevisiae*, in a wet process [24]. The pH value decreased and was stable after 4 days of fermentation, corresponding to viable yeast cells. This phenomenon can be explained by the rationale that LAB can grow and produce lactic and acetic acids, leading to a pH drop during the initial stage of submerged fermentation. Afterward, low pH leads to swelling and degradation of mucilage polysaccharides from coffee beans. Furthermore, a low pH encourages the growth of yeasts and prevents other bacteria. Meanwhile, enzymes from yeasts, such as pectinase and polygalacturonase, are active at a low pH to disassociate the mucilage of green coffee beans [25,26].

### 3.2. TPC Content

The TPC contents obtained from 33 treatments of submerged fermentation are demonstrated and ranged from 331.36 to 490.59 µmol GAE/100 g sample. The ANOVA for a quadratic model of TPC content plotted by a response surface is shown in Supplementary Table S3, which was significantly fitted with *p* < 0.05. The TPC content was significantly influenced by the linear and quadratic terms of the incubation time and coffee weight, while the linear and quadratic terms of temperature and the interaction terms of variables did not significantly impact the TPC content. The coefficient of determination (R-squared) value of the TPC content was 0.9103, implying that 91.03% of the TPC content can be fitted to the model. Additionally, the lack of fit value was insignificant, represented by *p* > 0.05 (1.000). This insignificant value can be explained by the fact that the model adequately describes the functional relationship between the experimental factors and the response variable. The regression equation for the TPC content was given as an Equation (3), where *X*_1_ is the temperature (°C), *X*_2_ is the incubation time (days), and *X*_3_ is the coffee weight (g).

(3) $$Y=432.29-0.86X_{1}+55.4X_{2}+11.21X_{3}+13.88X_{1}^{2}-47.72X_{2}^{2}+18.93X_{3}^{2}-3.75X_{1}X_{2}+0.38X_{1}X_{3}-4.93X_{2}X_{3}$$

**Figure 1:**
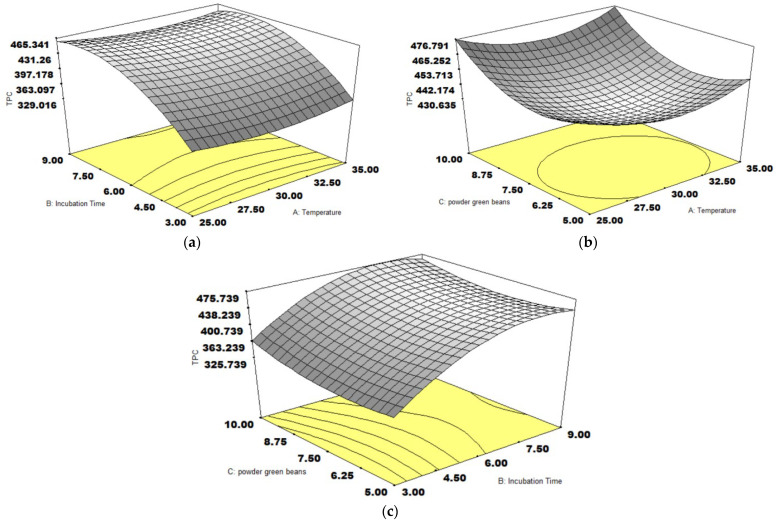
The response surface plots of three variables on TPC content (µmol GAE/100 g), temperature and incubation time (**a**), temperature and coffee weight (**b**), incubation time and coffee weight (**c**).

The response surface plots of TPC contents are displayed in Figure 1. The TPC content was dominantly influenced by the incubation time and coffee weight. The increase in incubation time promoted the TPC content (Figure 1a,c). This effect was consistent with the result reported by Bressani, Batista, Ferreira, Martinez, Simão, Dias and Schwan [26]. The extended incubation time of green coffee fermentation can stimulate the TPC content from different yeast treatments of self-induced anaerobic fermentation. The highest TPC content was derived from the fermentation of the yeast combination, including *S. cerevisiae*, *Candida parapsilosis*, and *Torulaspora delbrueckii*, at a 294.73 mg GAE/g sample. The enzymatic activity of yeasts can release some phenolic compounds from coffee fiber, leading to an increased TPC content [27]. In addition to yeasts, the effect of the incubation time was also observed in LAB. Extending the incubation time to 168 h can enhance the TPC content, approximately 5 mg GAE/g sample from inoculation of *L. plantarum* TISTR 543 [28]. However, the impact of coffee weight was inconsistent with the study of Myo, Nantarat, and Khat-Udomkiri [28]. The coffee concentration negatively impacted the TPC content. Three coffee parts in ten water parts provided a 3.34 mg GAE/g sample, superior to the 0.98 mg GAE/g sample of seven parts of coffee. Meanwhile, this study shows that a rise in the coffee weight increased the TPC content (Figure 1b,c). On the other hand, the temperature did not affect the TPC content, as shown in Figure 1a,b.

The optimal condition for submerged fermentation of green coffee beans was a temperature of 35 °C, an incubation time of 7.21 days, and 10 g of coffee weight (Table 1). The composite desirability of this condition was 0.846. The composite desirability ranged from zero to one, with one score representing the ideal case. On the contrary, zero value defines that one or more responses are outside their acceptable limits [29]. Hence, the composite desirability of this prediction was quite satisfied. The predicted content of TPC from this condition was 486.17, near the validated value of 480.25 ± 2.68 (Table 1), and the predicted error had a low value of −1.2%. The TPC content of the optimal submerged fermentation was higher than that of the solid-state fermentation (SSF), reporting 284.77 mg GAE/100 g sample [16].

### 3.3. ABTS Activity

Among all antioxidant activities, ABTS activity showed a potential value superior to DPPH and FRAP activities in the same unit. The lowest and highest values of ABTS activity were 300.5 and 896.22 µmol TE/g samples. ANOVA for the response surface quadratic model of ABTS is indicated in Supplementary Table S4. The model was significantly fitted because of *p* < 0.05, whereas the F-value was 23.69. Two dependent variables, consisting of incubation time and coffee weight, affected the linear and quadratic terms of ABTS activity, but temperature (*X*_1_) influenced only the linear term. The interaction terms of X_1 × 2_ and X_2 × 3_ were significantly involved in ABTS activity. The R-squared value of ABTS activity was high, representing the model fitted 97.82% of the ABTS. Furthermore, the experimental factors and the response variable were strongly correlated, which was described by the insignificance of lack of fit at *p* > 0.05 (0.0565). The regression equation for ABTS activity is shown as an Equation (4).

(4) $$Y=630.75+81.81X_{1}+75.93X_{2}+28.58X_{3}\;-26.65X_{1}^{2}\;-135.28X_{2}^{2}+83.09X_{3}^{2}+61.88X_{1}X_{2}+6.82X_{1}X_{3}+45.97X_{2}X_{3}$$

**Figure 2:**
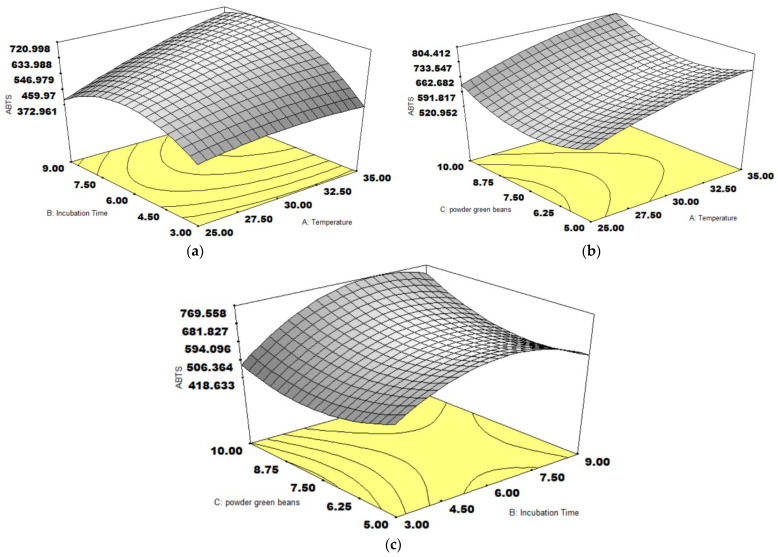
The response surface plots of three variables on ABTS activity (µmol TE/g), temperature and incubation time (**a**), temperature and coffee weight (**b**), incubation time, and coffee weight (**c**).

The response surface plots of ABTS activity shown in Figure 2 displayed three variables impacting ABTS activity. The raised temperature promoted ABTS activity, as observed in Figure 2a,b, and a higher coffee weight increased ABTS activity (Figure 2b,c). In contrast, the optimal incubation time was in a range of 6–7.5 days (Figure 2a,c). The influence of the incubation time was mentioned by Myo, Nantarat, and Khat-Udomkiri [28]. The ABTS activity increased to 4.91 mg TE/g sample before it decreased to 3.79 mg TE/g sample at 120 h of incubation time. The decrease in ABTS activity after 7.5 h of incubation time was explained by the LAB metabolism. *L. plantarum* can metabolize significant phenolic acids, such as chlorogenic acid and caffeic acid, through phenolic acid decarboxylation, reducing ABTS activity over the period [30]. However, the fermentation of *L. plantarum* represented that the ABTS scavenging activity decreased from 70.4% at 24 h of incubation to 57.02% before returning to about the same level at 72 h. The increase in ABTS activity might be related to the joint contribution of multiple microbial starters, leading to enhanced antioxidant activities [31]. Compared to the predicted ABTS activity (856.538 µmol TE/g sample), the validated value was more overestimated than the validated value, with the predicted error of −14.7%. The validation was 725.71 ± 0.71 µmol TE/g sample (Table 1). The ABTS at the optimal condition was much larger than that of the SSF at 422.54 µmol TE/g sample [16].

### 3.4. DPPH Activity

The DPPH activities (61.81–207.72 µmol TE/g sample) from various submerged fermentation conditions are shown in Supplementary Table S2. The model was significantly fitted (*p* < 0.05), as presented in the ANOVA for the response surface quadratic model (Supplementary Table S5). The DPPH activity was significantly impacted by the linear terms of temperature (X_1_) and coffee weight (X_3_), while quadratic terms of temperature (X_1_^2^) and incubation time (X_2_^2^) impacted DPPH activity. Additionally, all interaction terms were significant to the model (*p* > 0.05). The R-squared value of DPPH activity was 0.9057, accepted that 90.57% of DPPH activity can be fitted to the model. The fitted model can be also represented by *p* > 0.05 of lack of fit (0.2251). The regression equation for DPPH activity was demonstrated as an Equation (5).

(5) $$Y=147.89+7.31X_{1}+2.45X_{2}+15.12X_{3}+20.87X_{1}^{2}-\;51.04X_{2}^{2}+2.97X_{3}^{2}+7.62X_{1}X_{2}+8.21X_{1}X_{3}+12.01X_{2}X_{3}$$

**Figure 3:**
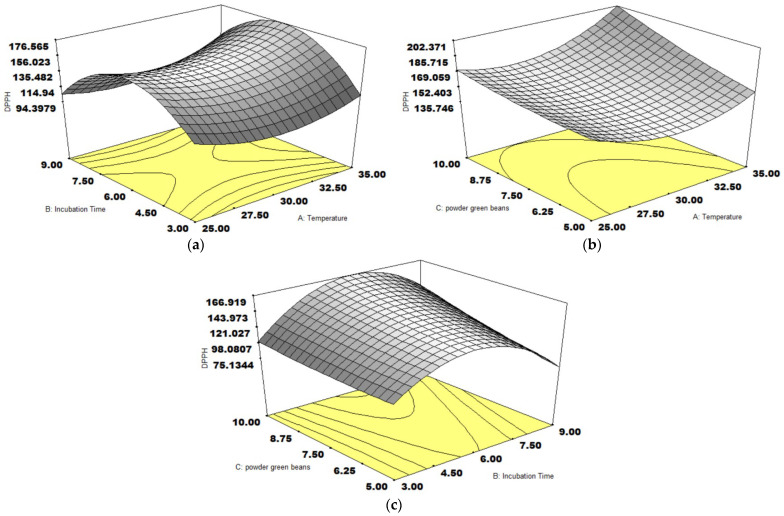
The response surface plots of three variables on DPPH activity (µmol TE/g), temperature and incubation time (**a**), temperature and coffee weight (**b**), incubation time and coffee weight (**c**).

The surface plots of DPPH activity can be visualized in Figure 3. Figure 3a,b displays that the increased temperature enhanced DPPH activity. Likewise, the gained coffee weight enhanced DPPH activity, as shown in Figure 3b,c. The temperature impact was also observed in the simultaneous aeration fermentation of coffee pulp using LAB. The optimal temperature to achieve the maximum DPPH activity of cascara was around 32 °C higher than 27 °C, depending on the incubation time [32]. 

The optimal condition for submerged fermentation specified in Table 1, a temperature of 35 °C, an incubation time of 7.21 days, and 10 g of coffee weight (0.846 scores of composite desirability) had 202.969 µmol TE/g sample of the predicted value. This value was higher than the validation (164.15 ± 2.40 µmol TE/g sample), and the predicted error was −19.7%. The experimental DPPH activity of optimal submerged fermentation was lower than that of SSF, reporting 191.21 µmol TE/g sample [16]. The DPPH activity was a significant measure of fermented coffee beans because this method is based on single electron transfer (SET) and hydrogen atom transfer (HAT) mechanisms [33]. However, the DPPH assay is based on a radical dissolved in organic media applied to hydrophobic antioxidant systems, while ABTS activity can detect hydrophilic and lipophilic antioxidant systems [34]. Thus, the values of ABTS activity were superior to the DPPH assay.

### 3.5. FRAP Activity

The FRAP activities derived from different submerged-fermentation conditions were the lowest at 276.24 µmol TE/g sample, whereas the highest FRAP activity was at 541.59 µmol TE/g sample. The ANOVA for the response surface quadratic model of FRAP activity was significantly fitted with *p* < 0.05 (Supplementary Table S6). The FRAP activity was significantly impacted by the linear terms of the incubation time (X_2_), coffee weight (X_3_), and the quadratic term of temperature (X_1_^2^), while the interaction terms were significant with X_1 × 3_ and X_2 × 3_. The R-squared value of FRAP activity was fitted to the model at 0.9060, and the lack of fit value was insignificant at 0.4132 (*p* > 0.05). The regression equation for FRAP activity is represented by an Equation (6).

(6) $$Y=355.36+5.44X_{1}+14.08X_{2}+57.83X_{3}+44.18X_{1}^{2}\;-3.9X_{2}^{2}+10.99X_{3}^{2}+17.8X_{1}X_{2}-41.04X_{1}X_{3}-36.45X_{2}X_{3}$$

**Figure 4:**
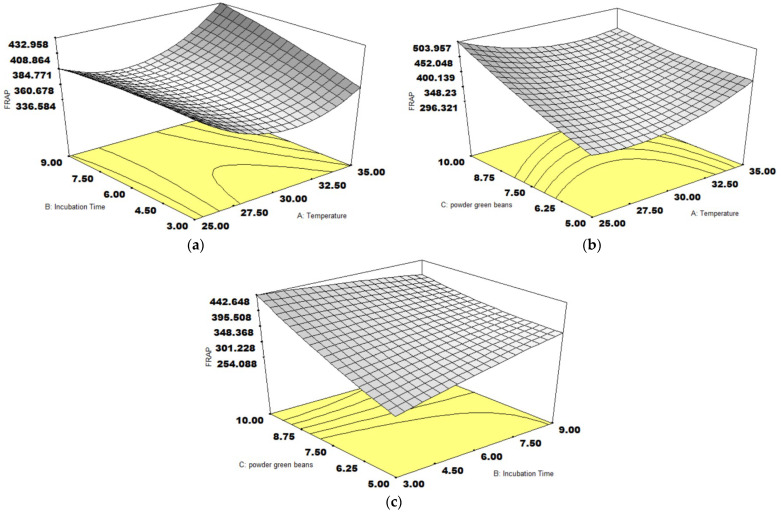
The response surface plots of three variables on FRAP activity (µmol TE/g), temperature and incubation time (**a**), temperature and coffee weight (**b**), incubation time, and coffee weight (**c**).

The response surface graph of FRAP activity is visualized in Figure 4. The incubation time and coffee weight were significant effects on FRAP activity. The extended incubation time encouraged FRAP activity as presented by Figure 4a,c, which was consistent with the effect of coffee weight and also promoted (Figure 4b,c). The levels of coffee antioxidants were referred to in terms of substrate concentration. The available glucose during fermentation induces the metabolism of nicotinamide adenine dinucleotide (NAD+) instead of the metabolic activity of phenolic acids [30]. This effect is related to the mono- or di-saccharide content in fermented coffee beans [35]. Accordingly, the extended coffee weight reduced phenolic acid metabolism and generated increased antioxidant activities through mucilage disassociation.

The optimal conditions (a temperature of 35 °C, an incubation time of 7.21 days, and 10 g of coffee weight) predicted the FRAP activity of 430.775. This prediction was slightly underestimated to the experimental value of 443.60 ± 8.19 µmol TE/g sample (Table 1). The predicted error was 3.0%, and the composite desirability was 0.846. This FRAP activity was superior to that of the SSF (272.11 µmol TE/g sample) [16]. 

### 3.6. Non-Targeted Metabolites

**Figure 5:**
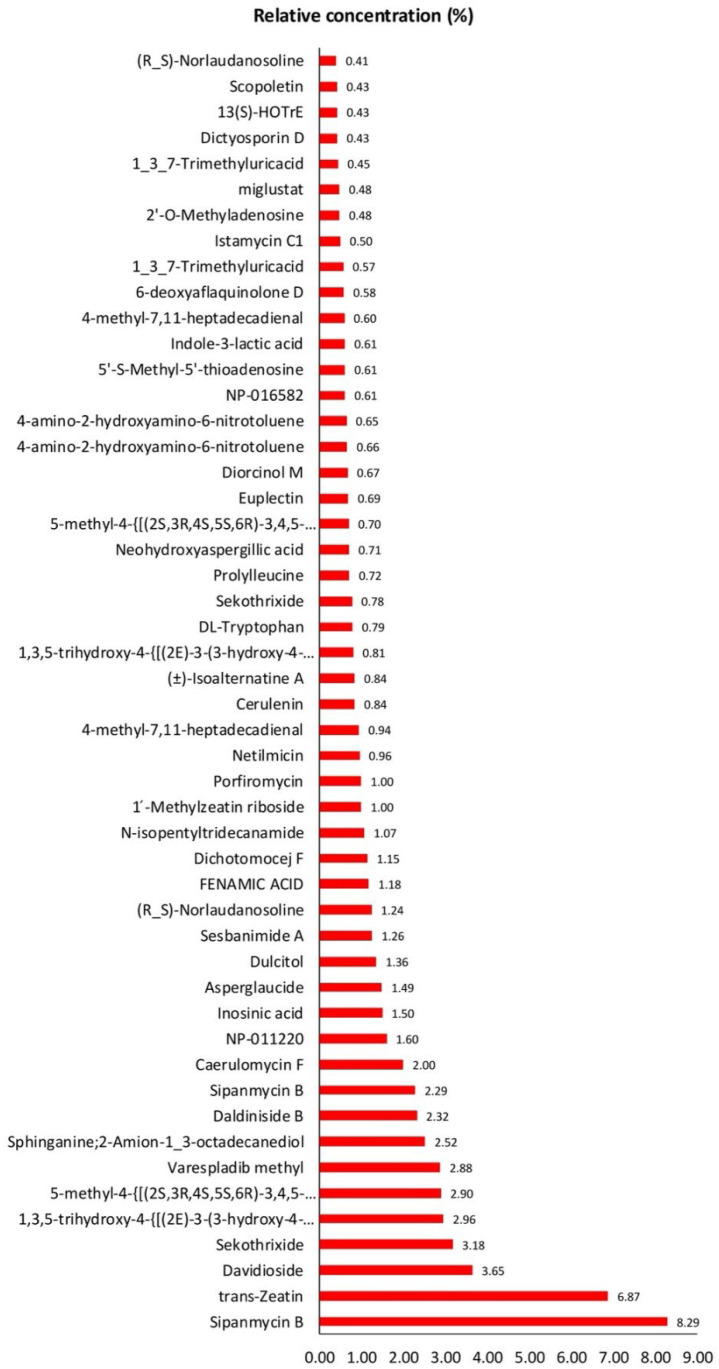
The top 50 untargeted metabolic compounds found in fermented green Robusta coffee beans by the microbial cocktails.

To investigate the metabolic profile of the FGCB extract using microbial cocktails, the 509 non-targeted metabolites were explored. The top 50 metabolic compounds are shown in Figure 5. The predominant metabolites observed in this fermentation aligned with those typically found in plants, including amino acids, fatty acids, lipids, and phenolic compounds. Among these, phenolic compounds were particularly abundant, suggesting a potential for enhanced antioxidant properties in fermented coffee beans. Particularly, 1,3,5-trihydroxy-4-{[(2E)-3-(3-hydroxy-4-methoxyphenyl)prop-2-enoyl]oxy}cyclohexane-1-carboxylic acid, 5-methyl-4-{[(2S,3R,4S,5S,6R)-3,4,5-trihydroxy-6-(hydroxymethyl)oxan-2-yl]oxy}-2H-chromen-2-one, Neohydroxyaspergillic acid, Diorcinol M, and Indole-3-lactic acid were highly abundant at 2.96%, 2.88%, 0.71%, 0.67%, and 0.61%, respectively. Phenolic compounds are known to exhibit antioxidant activity and promote several health benefits [36,37]. Notably, Indole-3-lactic acid, a metabolite of tryptophan produced by probiotics, serves as an anti-inflammatory in the gut [38].

### 3.7. Physical Properties of Ultrasonic-Treated Cherry Coffee

**Figure 6:**
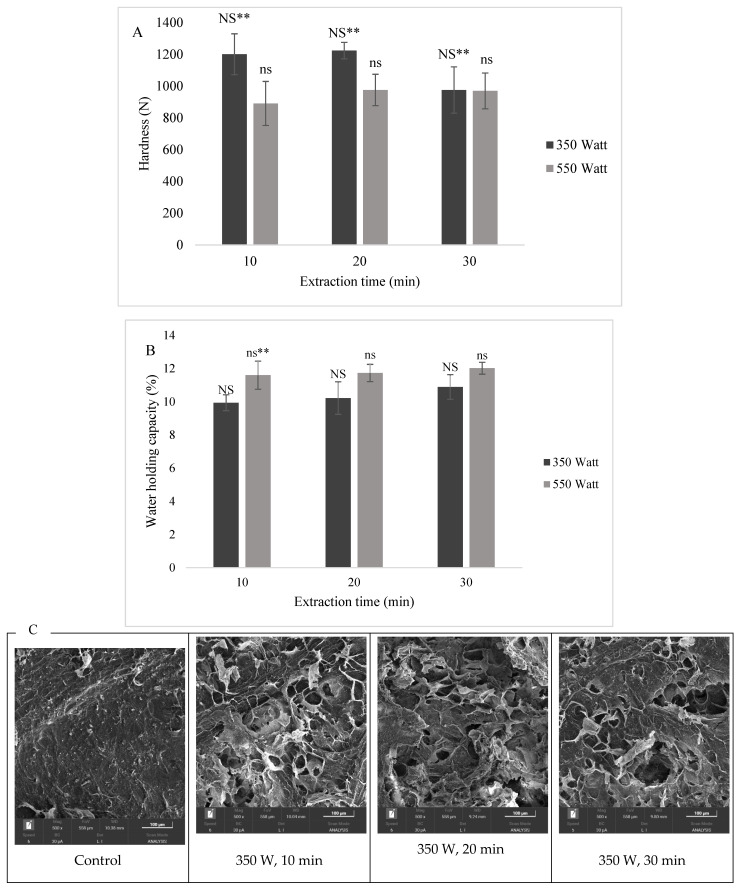

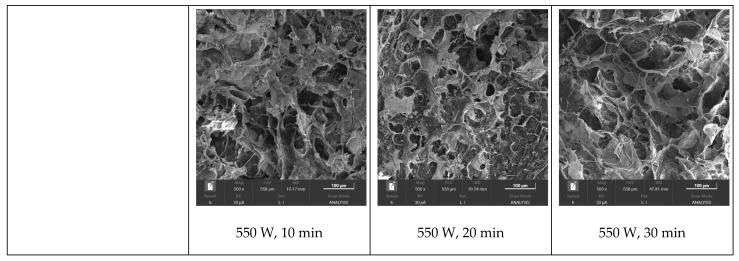
Effect of ultrasound power and treatment time on hardness (**A**), WHC (**B**) and morphology by scanning SEM (**C**) of coffee cherry. Note: ns indicates a non-significant difference between infusion time in a control sample (*p* > 0.05). NS indicates a non-significant difference between infusion time in an infused sample (*p* > 0.05). ** indicates a significant difference between control and infused sample at the same infusion time (*p* < 0.05).

The effect of ultrasonic power and treatment time on the WHC of coffee cherries is illustrated in Figure 6A. The results indicate that coffee cherries treated with higher ultrasonic power (550 W) exhibited significantly greater WHC compared to those treated at a lower power level (350 W) (*p* < 0.05). These findings align with previous research demonstrating that ultrasonic treatment enhances the water absorption and retention capacity of plant matrices by modifying their microstructure and porosity [39,40].

The improvement in WHC observed in this study corresponds with changes in texture properties after ultrasonic treatment. Specifically, an increase in ultrasonic power resulted in a reduction in hardness, suggesting that the ultrasonic waves disrupted the structural integrity of the coffee cherry matrix (Figure 6B). This effect can be attributed to acoustic cavitation, where the rapid formation and collapse of microscopic bubbles generate localized high pressure, leading to cell wall disruption and increased porosity [41]. However, the treatment time did not significantly impact either WHC or hardness (*p* > 0.05), indicating that ultrasonic intensity plays a more dominant role than exposure duration in modifying these properties. Mechanistically, the cavitation pressure generated by ultrasonic waves induces vapor pressure changes within the coffee cherry cells, leading to a breakdown of plant cell walls. This structural alteration enhances water absorption, increases the porosity of the matrix, and lowers its mechanical resistance, thereby reducing hardness and improving WHC. Similar effects have been reported in studies on ultrasonically treated fruits and seeds, where cavitation-induced cell wall disruption enhances water diffusion and retention [42,43].

Further evidence of structural modifications caused by ultrasonic treatment can be observed in the morphology of coffee cherries, as illustrated in Figure 6C. The internal structure of the ultrasonically treated coffee cherry exhibits increased porosity and a more open matrix, consistent with previous findings in food materials subjected to ultrasonic processing [44]. These changes have significant implications for coffee cherry processing, as improved WHC may influence drying kinetics, extraction efficiency, and overall quality attributes. The ultrasonic-treated coffee cherry at the power of 550 W for 10 min was used to prepare the coffee cherry for vacuum impregnation in the next part.

### 3.8. Vacuum Impregnation

**Figure 7:**
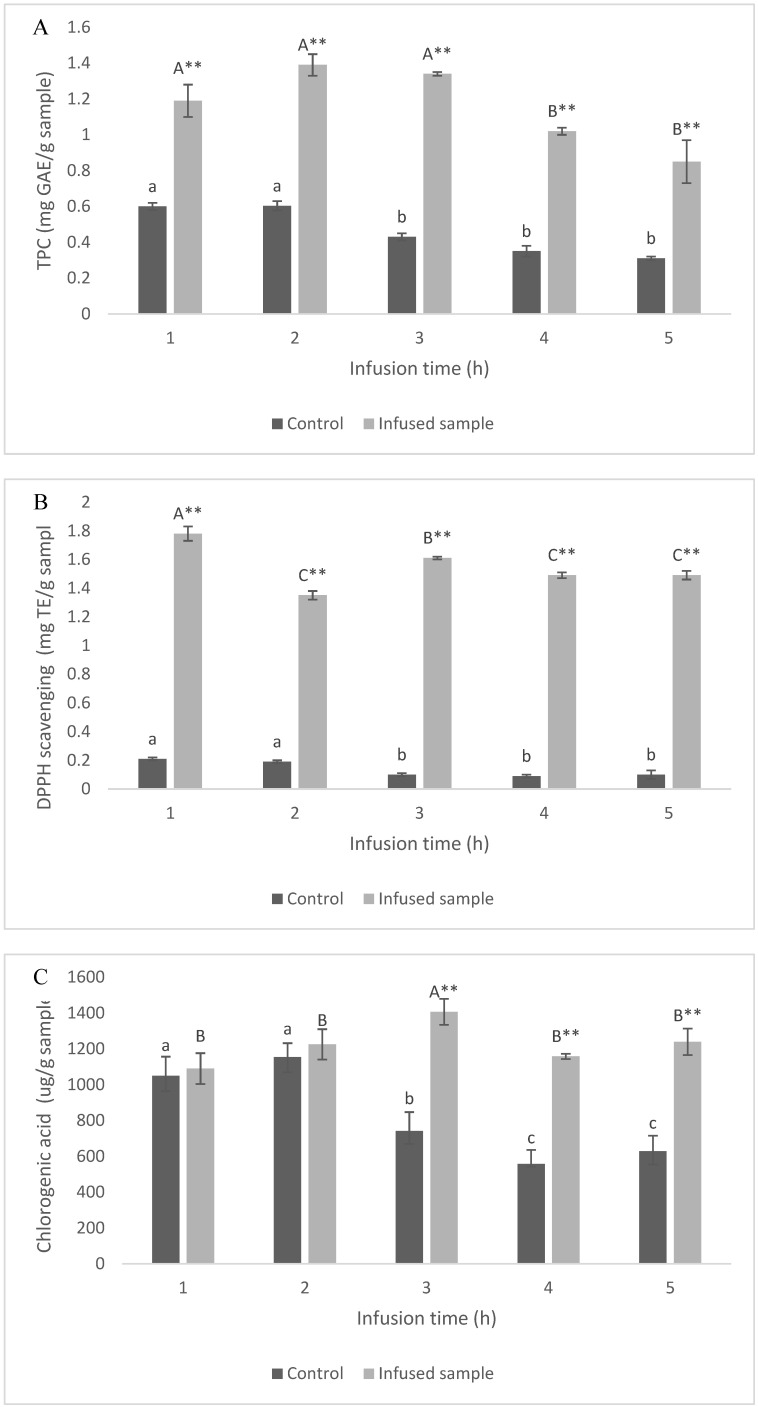
Effect of ultrasound power and treatment time on ABTS scavenging activity (**A**), DPPH scavenging activity (**B**) and chlorogenic acid (**C**) of coffee cherry. Note: a–c indicates a significant difference between infusion time in control sample (*p* < 0.05). A–C indicates a significant difference between infusion time in infused sample (*p* < 0.05). ** indicates a significant difference between control and infused sample at the same infusion time (*p* < 0.05).

The fermented green Robusta coffee beans were vacuum impregnated into ultrasonic-treated coffee cherry at different infusion times. The total phenolic content in coffee cherry after impregnation is shown in Figure 7A. The results indicate that applying fermented green Robusta coffee beans increased total phenolic content in cherry coffee at 1–3 h of impregnation; then, the phenolic content was decreased. Total phenolic content in the coffee cherry significantly increased from 0.6 ± 0.02 mg GAE/g to 1.19 ± 0.09 mg GAE/g after 1 h infusion (*p* < 0.05). However, after 3 h of infusion, the total phenolic content in the infused coffee cherry decreased from 1.19 ± 0.09 mg GAE/g to 0.85 ± 0.12 mg GAE/g. The phenolic content in infused coffee cherries is in line with the antioxidant activity of the sample. The DPPH scavenging activity of the infused sample showed antioxidant activity at 1.78 ± 0.1205 mg TE/g, significantly higher than 0.21 ± 0.01 mg TEGAE/g in the control sample after 1 h of infusion vacuum impregnation (*p* < 0.05). The results indicate that vacuum impregnation effectively facilitated the infusion of bioactive compounds from fermented green Robusta coffee beans into the coffee cherry’s porous structure, particularly after ultrasonic treatments. This process enhances the transfer and retention of beneficial phytochemicals, such as polyphenols, chlorogenic acids, and flavonoids, which contribute to the antioxidant and health-promoting properties of coffee. The combination of ultrasonic treatment and vacuum impregnation improves cell wall permeability, allowing for deeper penetration of bioactive compounds. These findings highlight the potential of advanced processing techniques in enriching coffee cherries with functional ingredients, ultimately enhancing their nutritional and functional value [45]. The results are supported by the study showing that a combined vacuum impregnation and ultrasound were proposed as an alternative method to improve the infusion of ascorbic acid in berry fruit [46].

The result can provide an explanation of how the impregnation may cause the loss of bioactive compounds. Chlorogenic acid is the most abundant compound found in coffee cherry and the fermented green coffee bean. After 3 h of vacuum impregnation, the chlorogenic acid content in the control sample was significantly decreased (*p* > 0.05). However, the chlorogenic acid content in the vacuum-impregnated sample was significantly higher than in the control sample (*p* < 0.05). This statement suggests that prolonged vacuum impregnation may lead to the release, rather than infusion, of bioactive compounds from the material. This phenomenon can be attributed to the extended exposure to vacuum conditions, which might cause structural degradation of plant tissues, leading to the leaching of intrinsic bioactive compounds into the impregnation medium. Research indicates that the efficiency of vacuum impregnation is influenced by factors such as vacuum pressure, impregnation time, and the physical properties of the plant matrix. For instance, excessive vacuum pressure or prolonged impregnation periods can disrupt cell integrity, resulting in the loss of native bioactive compounds [47]. Therefore, optimizing vacuum impregnation parameters is crucial to ensure the effective infusion of desired compounds while minimizing the unintended release of native bioactives.

Ultrasonic treatment resulted in a highly porous structure of the coffee cherry, leading to an increased WHC. These parameters significantly influence the transfer mechanisms of bioactive compounds from the FGCB to the coffee cherry during the vacuum impregnation process. The porosity of the structure plays a critical role by creating channels that facilitate the infiltration of solutions during vacuum impregnation, enhancing mass transfer efficiency. Greater porosity increases the permeability of the coffee cherry, enabling more rapid and uniform penetration of bioactive compound solutions [48]. WHC, on the other hand, reflects the ability of food materials to retain water or other liquid solutions. A high WHC indicates the potential of the coffee cherry to absorb and bind bioactive compounds from fermented green coffee solutions, thereby influencing the distribution and retention of nutrient compounds [49]. Together, these factors determine the efficiency of vacuum impregnation in transferring solutes into the matrix of the coffee cherry, ultimately impacting the overall quality and functionality of the final product.

The enhanced efficiency of vacuum impregnation significantly improves the infusion of bioactive compounds and augments the functional properties of coffee cherries. Ultrasonic treatment induces structural modifications, including increased porosity and surface area, which facilitate the penetration of bioactive compounds into plant matrices. Coffee cherries subjected to ultrasonic treatment demonstrated superior absorption capacity following vacuum impregnation with a submerged fermented green coffee bean solution. This process effectively introduced bioactive compounds, primarily chlorogenic acid, resulting in an elevated TPC and enhanced antioxidant capacity, as measured by ABTS, DPPH, and FRAP assays. These improvements are attributed to the optimization of mass transfer efficiency and the robust retention of active compounds within the porous matrix. Consequently, the fortified coffee cherry product exhibited enhanced functionality and biological activity.

## 4. Conclusions

The submerged fermentation of Robusta green coffee beans using selective microbial starters, such as *Hanseniaspora osmophila*, *Hanseniaspora vineae*, *Schizosaccharomyces osmophilus*, and *Saccharomyces cerevisiae*, including *Lactobacillus plantarum*, demonstrated the potential antioxidant production. The optimal conditions of three parameters, temperature, incubation time, and coffee weight, were 35 °C, 7.21 days, and 10 g, respectively. This condition offered TPC content and activities of ABTS, DPPH, and FRAP assays were 480.25 µmol GAE/100 g, 725.71, 164.15, and 443.60 µmol TE/g samples, respectively. Almost all these values were higher than those of the SSF of the previous study, except for DPPH activity. However, this research still lacks metabolic profiles of fermented coffee beans, which will be further studied in the following study. The vacuum impregnation method has been demonstrated as an effective technique for enriching coffee cherries with bioactive compounds extracted from fermented coffee beans, particularly when combined with ultrasonic pre-treatment. This process significantly enhances the infusion of key phytochemicals, improving the functional properties of the final product. After a 3-h vacuum infusion, the coffee cherry observed a substantial increase in total phenolic content, antioxidant activity, and chlorogenic acid concentration, indicating the successful incorporation of bioactive compounds. These findings highlight the potential of vacuum impregnation as a novel processing technique for fortifying plant-based food matrices with bioactive compounds, paving the way for developing functional food products with superior nutritional value and health-promoting properties [50]. The combination of vacuum impregnation and ultrasonic treatment presents a promising approach for enhancing the bioavailability and stability of beneficial phytochemicals in food applications.

## Figures and Tables

**Table 1:** Validation test results using optimum values of variables for the developed model.

	Temperature (°C)	Incubation Time (Days)	Coffee Weight (g)	TPC (µmol GAE/100 g)	ABTS (µmol TE/g)	DPPH (µmol TE/g)	FRAP (µmol TE/g)
Predicted Optimal condition	35	7.21	10	486.17	851.049	202.969	430.775
Experimental validation	35	7.21	10	480.25 ± 2.68	725.71 ± 0.71	164.15 ± 2.40	443.60 ± 8.19
Predicted error (%)	-	-	-	−1.2	−14.7	−19.1	3.0

## Data Availability

The data presented in this study and supporting data are available on request from the corresponding author.

## Supplementary Materials

The following supporting information can be downloaded at: https://www.mdpi.com/article/10.3390/foods14071165/s1, Table S1: The microbial counts and pH of submerged fermentation of green coffee bean; Table S2: The results of TPC content and antioxidant activities of submerged fermentation of green bean coffee; Table S3: ANOVA for a quadratic model of TPC content; Table S4: ANOVA for a quadratic model of ABTS content; Table S5: ANOVA for a quadratic model of DPPH activity; Table S6: ANOVA for a quadratic model of FRAP activity.

## References

[1] Erskine E., Gültekin Subaşı B.s.r., Vahapoglu B., Capanoglu E. (2022). Coffee phenolics and their interaction with other food phenolics: Antagonistic and synergistic effects. ACS Omega.

[2] Mostofsky E., Rice M.S., Levitan E.B., Mittleman M.A. (2012). Habitual coffee consumption and risk of heart failure: A dose-response meta-analysis. Circ. Heart Fail..

[3] Reis C.E., Dórea J.G., da Costa T.H. (2019). Effects of coffee consumption on glucose metabolism: A systematic review of clinical trials. J. Tradit. Complement. Med..

[4] Ferreira L.J.C., de Souza Gomes M., de Oliveira L.M., Santos L.D. (2023). Coffee fermentation process: A review. Food Res. Int..

[5] de Melo Pereira G.V., da Silva Vale A., de Carvalho Neto D.P., Muynarsk E.S., Soccol V.T., Soccol C.R. (2020). Lactic acid bacteria: What coffee industry should know?. Curr. Opin. Food Sci..

[6] Lee L.W., Cheong M.W., Curran P., Yu B., Liu S.Q. (2015). Coffee fermentation and flavor–An intricate and delicate relationship. Food Chem..

[7] Ludwig I.A., Clifford M.N., Lean M.E., Ashihara H., Crozier A. (2014). Coffee: Biochemistry and potential impact on health. Food Funct..

[8] Dziki D., Gawlik-Dziki U., Pecio Ł., Różyło R., Świeca M., Krzykowski A., Rudy S. (2015). Ground green coffee beans as a functional food supplement–Preliminary study. LWT-Food Sci. Technol..

[9] Espíndola K.M.M., Ferreira R.G., Narvaez L.E.M., Silva Rosario A.C.R., Da Silva A.H.M., Silva A.G.B., Vieira A.P.O., Monteiro M.C. (2019). Chemical and pharmacological aspects of caffeic acid and its activity in hepatocarcinoma. Front. Oncol..

[10] Toledo P.R., Pezza L., Pezza H.R., Toci A.T. (2016). Relationship between the different aspects related to coffee quality and their volatile compounds. Compr. Rev. Food Sci. Food Saf..

[11] Wang C., Sun J., Lassabliere B., Yu B., Zhao F., Zhao F., Chen Y., Liu S.Q. (2019). Potential of lactic acid bacteria to modulate coffee volatiles and effect of glucose supplementation: Fermentation of green coffee beans and impact of coffee roasting. J. Sci. Food Agric..

[12] Martinez S.J., Bressani A.P.P., Dias D.R., Simão J.B.P., Schwan R.F. (2019). Effect of bacterial and yeast starters on the formation of volatile and organic acid compounds in coffee beans and selection of flavors markers precursors during wet fermentation. Front. Microbiol..

[13] Zhang S.J., De Bruyn F., Pothakos V., Contreras G.F., Cai Z., Moccand C., Weckx S., De Vuyst L. (2019). Influence of various processing parameters on the microbial community dynamics, metabolomic profiles, and cup quality during wet coffee processing. Front. Microbiol..

[14] Lee L.W., Cheong M.W., Curran P., Yu B., Liu S.Q. (2016). Modulation of coffee aroma via the fermentation of green coffee beans with Rhizopus oligosporus: I. Green coffee. Food Chem..

[15] Lee L.W., Tay G.Y., Cheong M.W., Curran P., Yu B., Liu S.Q. (2017). Modulation of the volatile and non-volatile profiles of coffee fermented with Yarrowia lipolytica: I. Green coffee. LWT.

[16] Therdtatha P., Jareontanahun N., Chaisuwan W., Yakul K., Paemanee A., Manassa A., Moukamnerd C., Phimolsiripol Y., Sommano S.R., Seesuriyachan P. (2023). Production of functional Arabica and Robusta green coffee beans: Optimization of fermentation with microbial cocktails to improve antioxidant activity and metabolomic profiles. Biocatal. Agric. Biotechnol..

[17] Chavan P., Sharma P., Sharma S.R., Mittal T.C., Jaiswal A.K. (2022). Application of high-intensity ultrasound to improve food processing efficiency: A review. Foods.

[18] Akhoundzadeh Yamchi A., Yeganeh R., Kouchakzadeh A. (2022). Effect of ultrasonic pretreatment on drying kinetics and physio-mechanical characteristics of peach slices. J. Food Process Eng..

[19] Singleton V.L., Orthofer R., Lamuela-Raventós R.M. (1999). [14] Analysis of total phenols and other oxidation substrates and antioxidants by means of folin-ciocalteu reagent. Methods in Enzymology.

[20] Marc F., Davin A., Deglene-Benbrahim L., Ferrand C., Baccaunaud M., Fritsch P. (2004). Méthodes d’évaluation du potentiel antioxydant dans les aliments. Médecine/Sciences.

[21] Arulpriya P., Lalitha P., Hemalatha S. (2010). Antioxidant activities of the extracts of the aerial roots of Pothos aurea (Linden ex Andre). Der Pharma Chem..

[22] Li D., Li B., Ma Y., Sun X., Lin Y., Meng X. (2017). Polyphenols, anthocyanins, and flavonoids contents and the antioxidant capacity of various cultivars of highbush and half-high blueberries. J. Food Compos. Anal..

[23] Ballesteros L.F., Teixeira J.A., Mussatto S.I. (2014). Chemical, functional, and structural properties of spent coffee grounds and coffee silverskin. Food Bioprocess Technol..

[24] Bae H.M., Haile M., Kang W.H. (2022). Evaluation of antioxidant, organic acid, and volatile compounds in coffee pulp wine fermented with native yeasts isolated from coffee cherries. Food Sci. Technol. Int..

[25] Silva C.F. (2014). Microbial activity during coffee fermentation. Cocoa and Coffee Fermentations.

[26] Bressani A.P.P., Batista N.N., Ferreira G., Martinez S.J., Simão J.B.P., Dias D.R., Schwan R.F. (2021). Characterization of bioactive, chemical, and sensory compounds from fermented coffees with different yeasts species. Food Res. Int..

[27] Silva C.F., Vilela D.M., de Souza Cordeiro C., Duarte W.F., Dias D.R., Schwan R.F. (2013). Evaluation of a potential starter culture for enhance quality of coffee fermentation. World J. Microbiol. Biotechnol..

[28] Myo H., Nantarat N., Khat-Udomkiri N. (2021). Changes in bioactive compounds of coffee pulp through fermentation-based biotransformation using *Lactobacillus plantarum* TISTR 543 and its antioxidant activities. Fermentation.

[29] Amdoun R., Khelifi L., Khelifi-Slaoui M., Amroune S., Asch M., Assaf-Ducrocq C., Gontier E. (2018). The desirability optimization methodology; a tool to predict two antagonist responses in biotechnological systems: Case of biomass growth and hyoscyamine content in elicited Datura starmonium hairy roots. Iran. J. Biotechnol..

[30] Filannino P., Bai Y., Di Cagno R., Gobbetti M., Gänzle M.G. (2015). Metabolism of phenolic compounds by *Lactobacillus* spp. during fermentation of cherry juice and broccoli puree. Food Microbiol..

[31] Li Z., Teng J., Lyu Y., Hu X., Zhao Y., Wang M. (2018). Enhanced antioxidant activity for apple juice fermented with *Lactobacillus plantarum* ATCC14917. Molecules.

[32] Oktaviani L., Astuti D.I., Rosmiati M., Abduh M.Y. (2020). Fermentation of coffee pulp using indigenous lactic acid bacteria with simultaneous aeration to produce cascara with a high antioxidant activity. Heliyon.

[33] Liang N., Kitts D.D. (2014). Antioxidant property of coffee components: Assessment of methods that define mechanisms of action. Molecules.

[34] Floegel A., Kim D.-O., Chung S.-J., Koo S.I., Chun O.K. (2011). Comparison of ABTS/DPPH assays to measure antioxidant capacity in popular antioxidant-rich US foods. J. Food Compos. Anal..

[35] Wang C., Sun J., Lassabliere B., Yu B., Liu S.Q. (2020). Coffee flavour modification through controlled fermentations of green coffee beans by Saccharomyces cerevisiae and Pichia kluyveri: Part I. Effects from individual yeasts. Food Res. Int..

[36] Yust B.G., Wilkinson F., Rao N.Z. (2023). Variables affecting the extraction of antioxidants in cold and hot brew coffee: A review. Antioxidants.

[37] Lin D., Xiao M., Zhao J., Li Z., Xing B., Li X., Kong M., Li L., Zhang Q., Liu Y. (2016). An Overview of Plant Phenolic Compounds and Their Importance in Human Nutrition and Management of Type 2 Diabetes. Molecules.

[38] Meng D.I., Sommella E., Salviati E., Campiglia P., Ganguli K., Djebali K., Zhu W., Walker W.A. (2020). Indole-3-lactic acid, a metabolite of tryptophan, secreted by *Bifidobacterium longum* subspecies infantis is anti-inflammatory in the immature intestine. Pediatr. Res..

[39] Sun W., Shahrajabian M.H. (2023). Therapeutic Potential of Phenolic Compounds in Medicinal Plants-Natural Health Products for Human Health. Molecules.

[40] Zhou L., Wang L., Ding S., Liu D. (2020). Impact of ultrasound-assisted processing on food structure and functionality: A review. Critical Reviews in Food Science and Nutrition.

[41] García-Pérez J.V., Cárcel J.A., Benedito J., Mulet A. (2019). Ultrasonics and food processing: Recent advances and future perspectives. Trends Food Sci. Technol..

[42] Xue H., Tu Y., Zhang G., Xin X., Hu H., Qiu W., Ruan D., Zhao Y. (2021). Mechanism of ultrasound and tea polyphenol assisted ultrasound modification of egg white protein gel. Ultrasonics Sonochemistry.

[43] Chen H., Gao J., Zhang M., Adhikari B. (2018). Effect of ultrasonic treatment on hydration and quality properties of plant-based food materials. Food Chem..

[44] Jambrak A.R., Mason T.J., Lelas V., Herceg Z., Herceg I.L. (2020). Effects of ultrasound treatment on physicochemical properties of food products. Ultrasonics Sonochemistry.

[45] Jadhav H.B., Annapure U.S. (2021). Influence of high-intensity ultrasound processing on microstructure and quality parameters of plant-based foods. Ultrasonics Sonochemistry.

[46] Panayampadan A.S., Alam M.S., Aslam R., Kaur J. (2022). Vacuum impregnation process and its potential in modifying sensory, physicochemical and nutritive characteristics of food products. Food Eng. Rev..

[47] Mierzwa D., Szadzińska J., Gapiński B., Radziejewska-Kubzdela E., Biegańska-Marecik R. (2022). Assessment of ultrasound-assisted vacuum impregnation as a method for modifying cranberries’ quality. Ultrasonics Sonochemistry.

[48] Durán-Castañeda A.C., González-Moya S., Sánchez-Burgos J.A., Sáyago-Ayerdi S.G., Zamora-Gasga V.M. (2024). Applications of vacuum impregnation as a technology to incorporate functional components in vegetal matrices. Food Chem. Adv..

[49] Santarelli V., Neri L., Moscetti R., Di Mattia C.D., Sacchetti G., Massantini R., Pittia P. (2021). Combined use of blanching and vacuum impregnation with trehalose and green tea extract as pre-treatment to improve the quality and stability of frozen carrots. Food Bioprocess Technol..

[50] Zhao W., Yu D., Xia W. (2021). Vacuum impregnation of chitosan coating combined with water-soluble polyphenol extracts on sensory, physical state, microbiota composition and quality of refrigerated grass carp slices. Int. J. Biol. Macromol..

