# Supplementary material for: Enhanced Bioactive Coffee Cherry: Infusion of Submerged-Fermented Green Coffee Beans via Vacuum Impregnation
Source: Foods. 2025 Mar 27;14(7):1165. doi: 10.3390/foods14071165 (PMC11989068; doi:10.3390/foods14071165)
Supplement: Supplementary file 1 [file foods-14-01165-s001.zip › foods-3527286-supplementary.pdf]

Supplementary Materials

Table S1 The microbial counts and pH of submerged fermentation of green coffee bean
Table S2. The results of TPC content and antioxidant activities of submerged fermentation of green bean coffee
Table S3 ANOVA for a quadratic model of TPC content
Table S4 ANOVA for a quadratic model of ABTS activity
Table S5 ANOVA for a quadratic model of DPPH activity
Table S6 ANOVA for a quadratic model of FRAP activity

Table S1 The microbial counts and pH of submerged fermentation of green coffee bean

Run	Temperature (°C)	Incubation time (days)	Coffee weight (g)	Microbial counts (log cfu/g)		pH
				Yeasts	LAB	
1	25	6	10	5.61±0.01	3.57±0.07	4.41
2	30	6	7.5	4.78±0.01	3.57±0.07	4.48
3	35	6	7.5	3.50±0.02	3.50±0.02	3.77
4	35	3	10	4.41±0.10	4.48±0.01	4.77
5	35	9	5	5.69±0.03	3.39±0.05	4.31
6	25	3	5	3.61±0.01	4.67±0.02	4.76
7	30	6	5	3.57±0.06	3.50±0.02	4.48
8	30	9	10	5.51±0.01	4.54±0.02	4.51
9	30	3	7.5	4.54±0.04	4.62±0.01	4.86
10	30	6	7.5	3.61±0.01	3.49±0.02	4.50
11	25	9	7.5	5.84±0.04	4.71±0.01	3.30
12	35	6	10	4.47±0.02	3.57±0.07	3.80
13	25	9	10	3.65±0.02	3.50±0.05	4.33
14	30	6	7.5	4.62±0.01	3.57±0.07	4.53
15	30	6	7.5	3.49±0.02	3.56±0.06	4.38
16	30	6	7.5	3.57±0.05	3.58±0.02	4.53
17	25	6	5	3.42±0.08	3.50±0.02	4.36
18	35	9	7.5	3.45±0.02	3.67±0.05	4.50
19	30	3	10	5.63±0.11	5.79±0.15	4.91
20	30	9	5	3.50±0.02	3.79±0.01	3.66
21	35	3	5	4.62±0.06	4.00±0.04	4.82
22	25	3	7.5	3.76±0.01	4.94±0.03	4.94
23	35	9	10	3.57±0.07	3.57±0.02	3.56
24	30	3	5	4.52±0.01	4.57±0.01	4.78
25	30	6	10	5.68±0.05	3.61±0.01	4.52
26	35	3	7.5	3.60±0.01	3.49±0.02	4.84
27	30	6	7.5	3.69±0.02	3.61±0.01	4.51
28	30	9	7.5	4.84±0.01	3.91±0.01	4.51
29	25	9	5	3.82±0.03	3.65±0.02	3.89

30	35	6	5	3.51±0.02	3.61±0.01	3.93
31	30	6	7.5	4.53±0.04	3.61±0.01	4.51
32	25	3	10	4.67±0.04	4.55±0.01	4.90
33	25	6	7.5	3.54±0.02	3.57±0.01	4.42

Table S2. The results of TPC content and antioxidant activities of submerged fermentation of green bean coffee

Run	Temperature (°C)	Incubation time (days)	Coffee weight (g)	TPC (µmol GAE/100 g)	ABTS (µmol TE/g)	DPPH (µmol TE/g)	FRAP (µmol TE/g)
1	25	6	10	490.59±0.75	599.56±5.67	163.68±1.94	523.97±0.82
2	30	6	7.5	449.97±1.84	599.32±2.84	138.57±1.37	377.54±2.95
3	35	6	7.5	453.97±0.15	690.64±8.55	177.81±0.66	402.22±1.42
4	35	3	10	385.06±0.35	490.0±3.19	123.79±0.49	498.16±0.61
5	35	9	5	459.55±0.34	651.59±4.26	96.69±3.17	487.01±3.47
6	25	3	5	335.32±0.06	450.67±6.76	108.08±0.63	281.02±1.23
7	30	6	5	448.93±0.68	690.16±5.91	163.09±0.55	331.76±4.33
8	30	9	10	466.81±0.78	732.01±1.84	109.62±1.23	458.66±12.06
9	30	3	7.5	331.36±0.50	418.88±4.03	108.08±0.96	340.52±4.43
10	30	6	7.5	440.99±0.56	589.32±5.67	146.29±0.48	404.81±8.01
11	25	9	7.5	464.77±0.22	400.59±8.19	103.08±0.32	419.28±0.82
12	35	6	10	475.87±0.17	758.55±1.64	207.72±0.73	421.02±4.56
13	25	9	10	479.21±0.15	501.33±2.95	136.74±0.66	441.34±0.82
14	30	6	7.5	450.91±0.44	600.26±9.12	130.41±1.11	372.96±8.52
15	30	6	7.5	454.09±1.12	601.17±9.93	151.89±0.97	364.85±4.56
16	30	6	7.5	367.09±1.32	578.45±16.44	136.61±0.14	322.46±2.21
17	25	6	5	452.75±0.61	560.79±5.73	160.72±1.11	294.79±2.49
18	35	9	7.5	457.57±0.17	670.87±2.95	139.45±0.63	439.09±0.82
19	30	3	10	364.92±0.22	409.18±1.62	105.46±1.04	405.23±7.08
20	30	9	5	453.01±0.59	540.14±3.28	61.81±0.32	322.77±6.18
21	35	3	5	344.13±0.29	455.11±4.43	102.59±2.16	290.22±1.84
22	25	3	7.5	350.20±0.19	300.5±6.84	113.13±0.76	389.22±2.13
23	35	9	10	471.17±0.22	896.22±2.84	182.12±0.8	390.33±1.64
24	30	3	5	343.59±0.17	599.21±8.44	101.97±0.6	276.24±4.03
25	30	6	10	459.85±0.47	789.22±2.17	164.68±1.93	378.99±3.28
26	35	3	7.5	339.45±0.34	456.78±6.41	110.56±0.73	345.21±2.61
27	30	6	7.5	449.01±0.82	690.63±5.91	148.85±0.97	334.87±6.18
28	30	9	7.5	433.01±0.56	630.75±2.17	116.95±0.32	341.93±2.17
29	25	9	5	471.51±2.00	457.76±1.64	90.22±1.14	320.42±4.26
30	35	6	5	448.29±1.12	790.34±2.84	155.48±1.26	414.07±0.82
31	30	6	7.5	398.11±1.27	679.45±2.84	145.66±0.90	339.33±2.68
32	25	3	10	365.35±0.45	534.22±1.06	118.97±0.14	541.59±1.3
33	25	6	7.5	440.83±1.33	582.11±2.84	169.95±0.32	377.76±2.17

Table S3 ANOVA for a quadratic model of TPC content

Source	Sum of Square	df	Mean of square	F-value	Prob > F
Model	75445.89	9	8382.88	23.69	< 0.0001
X_1	13.29	1	13.29	0.038	0.8482
X_2	55252.38	1	55252.38	156.14	< 0.0001
X_3	2261.19	1	2261.19	6.39	0.0196
X_1^2	1399.08	1	1399.08	3.95	0.0600
X_2^2	16542.80	1	16542.80	46.75	< 0.0001
X_3^2	2603.00	1	2603.00	7.36	0.0131
X_1X_2	168.39	1	168.39	0.48	0.4979
X_1X_3	1.73	1	1.73	4.896E-003	0.9449
X_2X_3	291.71	1	291.71	0.82	0.3742
Lack of Fit	1226.52	17	72.15	0.047	1.0000
R^2	0.9103				
Adj R^2	0.8719				

Table S4 ANOVA for a quadratic model of ABTS activity

Source	Sum of Square	df	Mean of square	F-value	Prob > F
Model	4.799E+005	9	53318.66	104.82	< 0.0001
X_1	1.205E+005	1	1.205E+005	236.83	< 0.0001
X_2	1.038E+005	1	1.038E+005	204.01	< 0.0001
X_3	14706.73	1	14706.73	28.91	< 0.0001
X_1^2	5156.96	1	5156.96	10.14	0.0045
X_2^2	1.329E+005	1	1.329E+005	261.32	< 0.0001
X_3^2	50146.75	1	50146.75	98.58	< 0.0001
X_1X_2	45943.17	1	45943.17	90.32	< 0.0001
X_1X_3	558.09	1	558.09	1.10	0.3068
X_2X_3	25360.68	1	25360.68	49.86	< 0.0001
Lack of Fit	10238.61	17	602.27	5.43	0.0565
R^2	0.9782				
Adj R^2	0.9689				

Table S5 ANOVA for a quadratic model of DPPH activity

Source	Sum of Square	df	Mean of square	F-value	Prob > F
Model	28336.40	9	3148.49	22.41	< 0.0001
X_1	962.56	1	962.56	6.85	0.0161
X_2	107.82	1	107.82	0.77	0.3909

X_3	4114.28	1	4114.28	29.28	< 0.0001
X_1^2	3162.24	1	3162.24	22.51	0.0001
X_2^2	18924.75	1	18924.75	134.70	< 0.0001
X_3^2	64.27	1	64.27	0.46	0.5062
X_1X_2	697.24	1	697.24	4.96	0.0370
X_1X_3	808.71	1	808.71	5.76	0.0258
X_2X_3	1732.19	1	1732.19	12.33	0.0021
Lack of Fit	2671.02	17	157.12	2.25	0.2251
R^2	0.9057				
Adj R^2	0.8653				

Table S6 ANOVA for a quadratic model of FRAP activity

**Source**	**Sum of Square**	**df**	**Mean of square**	**F-value**	**Prob > F**
Model	1.225E+005	9	13613.11	22.49	< 0.0001
X_1	532.90	1	532.90	0.88	0.3588
X_2	3567.87	1	3567.87	5.89	0.0243
X_3	60203.34	1	60203.34	99.45	< 0.0001
X_1^2	14175.27	1	14175.27	23.42	< 0.0001
X_2^2	110.60	1	110.60	0.18	0.6734
X_3^2	877.14	1	877.14	1.45	0.2421
X_1X_2	3803.15	1	3803.15	6.28	0.0205
X_1X_3	20209.74	1	20209.74	33.39	< 0.0001
X_2X_3	15941.04	1	15941.04	26.33	< 0.0001
Lack of Fit	10859.36	17	638.79	1.38	0.4132
R^2	0.9060				
Adj R^2	0.8657				
